# Psychiatry

**Published:** 1905-10-28

**Authors:** 


### PSYCHIATRY.

(*Concluded from page* 10.)

**Somnolentia: a Symptom allied to Somnambulism.**—Dr. G. W. Taylor,[5] of the Boston Society of Psychiatry, has drawn attention to cases on the borderland of sanity and insanity, with characteristic symptoms of automatism and manifesting morbid aberrations of conduct—a subject of some medico-legal importance. Among the illustrative cases cited by Dr. Taylor (at the meeting of the Boston Society of Psychiatry held in February 1905) was that of a man of 31 years of age who for several years had been "annoyed by a certain dream-state in which he left his bed and performed various purposeless acts impelled by an impulse of fear over which he had no control." This had happened from one to several times a year and on such occasions he would find himself at a distance from his room in a strange place, having in his state of somnolentia or dream-walking passed through his bedroom window and down to the ground three storeys below by clinging to a large copper water-pipe attached to the wall on the outside of his room. He had many other such experiences. The point of special importance lay in the fact that he remembered perfectly the details of what happened between the time of leaving his bed and his final awakening some time after. He usually "woke from his condition of dream-walking suddenly and would then return to his bed without nervous perturbation and again quickly fall asleep." The condition indicates, adds Dr. Taylor, a curious modification of ordinary sleep-walking (somnambulism) in a person apparently free from hysteria or epileptic neurosis. In the discussion which followed the reading of this paper Dr. Dane suggested that persons in such states of automatism, combined with inhibition of the higher consciousness, might attempt to commit murder or an act of violence which suggested that they were under the influence of a definite morbid phobia or obsessional impulse. Dr. Hall said that the medico-legal and psychological problem presented by these cases showed how closely related are the states of consciousness in somnolentia (dream-walking) to the disaggregation and multi-personal manifestations of the ego in the higher forms of hysteria and psychical epilepsy, in the latter of which loss of the sense of personal identity has been known to occur and the patient has been found wandering from his home without knowing or being able to recollect his home, his friends, or even his own personal identity and his name. Pierre Janet, of Paris, has emphasised the splitting up or partial "disaggregation" of the constituents of the higher or conscious ego as an "explanation of hysterical states." "In the present case," says Dr. Hall, "we have a definite phobia, with an urgent obsessional idea or demand which is followed out—a systematised state of consciousness, if you please, marked by a dominant, if not a fulminating, excess in active attention . . . the normal interaction between self and circumstance—*i.e.*, experience—being in abeyance." In such morbid mental states, usually of temporary character, ordinary sensorial stimuli fail at first to arouse normal consciousness; later, impressions far more feeble disturb the sequence of thought of action, and "the patient suddenly returns to a full comprehension of himself as related to his surroundings." These cases do not present marked or obvious physical stigmata of degeneracy; they are to be regarded as only psychic abnormalities of an obsessional character, having a liability to periodic or irregular recurrence; they are very important, owing to the medico-legal question of responsibility which may be raised in the case of deeds done and crimes perpetrated by such persons on the borderland of sanity and insanity. Dream-walking of somnolentia thus has a practical bearing in its medico-legal as well as psychological aspects.

[5] Jour. of Nerv. and Ment. Dis., Aug. 1905.

